# Partnering with families to promote nutrition in cancer care: feasibility and acceptability of the PIcNIC intervention

**DOI:** 10.1186/s12904-018-0306-4

**Published:** 2018-03-20

**Authors:** Alex Molassiotis, Shelley Roberts, Hui Lin Cheng, Henry K. F. To, Po Shan Ko, Wang Lam, Yuk Fong Lam, Jessica Abbott, Deborah Kiefer, Jasotha Sanmugarajah, Andrea P. Marshall

**Affiliations:** 1 School of Nursing, The Hong Kong Polytechnic University, Hung Hom, Hong Kong; 2 National Centre of Research Excellence in Nursing, Menzies Health Institute Queensland, Griffith University, Gold Cost, Australia; 3 Department of Medicine, Haven of Hope Hospital, Tseung Kwan O, Hong Kong; 4 Dietetics Department, Haven of Hope Hospital, Tseung Kwan O, Hong Kong; 5 Gold Coast University Hospital, Gold Coast Health, Southport, Australia

**Keywords:** Nutrition, Intervention, Eating-related distress, Energy, Protein, Family caregiver, Advanced cancer

## Abstract

**Background:**

Malnutrition is frequent in patients with cancer, particularly those in advanced stages of the disease. The aim of the present study was to test the feasibility of a family-centred nutritional intervention, based on the Family Systems theory and past research.

**Methods:**

This was a single-arm trial assessing feasibility (eligibility, recruitment and retention rates); acceptability by patients, family caregivers and health professionals; intervention fidelity, and energy/protein intake (in one site only). Two sites were involved; one each in Australia (AUS) and Hong Kong (HK), with one site delivering the intervention to oncology patients receiving curative treatments in the hospital, and the other to advanced cancer patients in the home.

**Results:**

The sample included 53 patients (23 from AUS and 30 from HK), 22 caregivers (3 from AUS and 19 from HK) and 30 health professionals (20 from AUS and 10 from HK). Recruitment was difficult in the acute inpatient oncology care setting (AUS) and feasibility criteria were not met. Sufficient recruitment took place in the home care setting with advanced cancer patients in HK. Patients, family members and health professionals found the intervention helpful and acceptable, and patients and families indicated they would take part in the future in a similar study. Energy and protein intake improved from baseline to end of intervention (mean 22 kcal/kg/day to 26 and 0.9 g/kg/day to 1.0 respectively).

**Conclusion:**

The new intervention is feasible in a home setting when delivered to patients with advanced cancer, acceptable to patients and families, and has the potential to improve nutritional status in patients. A large randomised trial is warranted in the future.

**Electronic supplementary material:**

The online version of this article (10.1186/s12904-018-0306-4) contains supplementary material, which is available to authorized users.

## Background

Malnutrition is common in the hospital setting, affecting 20–50% of patients [1, 2], defined as “a state of nutrition in which a deficiency, excess or imbalance of energy, protein, and other nutrients causes measurable adverse effects on tissue/body form (body shape, size, and composition) and function, and clinical outcome” [3]. It results in increased risk of mortality [4] and complications such as pressure injury [5], falls [5, 6] and infections [7], and increased length of stay, hospital costs, and readmission rates [4]. A complex mix of factors relating to disease, food intake and the hospital environment contributes to the development of malnutrition in hospitalised patients.

Cancer patients are at particularly high risk of malnutrition due to metabolic, physiological, physical and psychological changes associated with cancer treatments and the disease itself. A range of nutrition-impacting symptoms such as poor appetite, nausea, vomiting, diarrhoea, chewing or swallowing problems and taste and smell changes are common in patients with cancer [3]. Severe malnutrition accounts for around 30% of cancer-related deaths overall; 30–50% of deaths in patients with gastrointestinal tract cancers and up to 80% of deaths in patients with advanced pancreatic cancer [8]. While there is little evidence to suggest that nutritional intake may improve survival, it can improve nutritional status and quality of life [3, 9]. Hence, strategies to improve the nutritional intake of patients with cancer are clearly warranted.

Previous research has shown that involving hospitalised patients in their nutrition care is an effective way of improving their energy and protein intake [9, 10]. Our data suggests that patients are likely to rely on their families for nutritional support and families often wish to be actively engaged in the patient’s nutrition care [11]. Actively involving patients and families in nutrition care during hospitalisation and beyond may be an effective way of improving nutrition delivery and intake in these high-risk patients.

The qualitative research and systematic review from our team has found that eating and weight-related problems are common in advanced cancer patients, but these symptoms extend beyond reduced food intake, also including physical, psychological, social and spiritual consequences [11–13]. We have also shown that patients often struggle with weight loss or being pressured by the family to eat, and feel they do not receive appropriate dietary advice, often resulting in self-managing their diet and weight loss [11, 13].

Nutritional care remains a challenging area in supportive cancer care. Dietary counselling is the most commonly used approach in clinical settings, with motivational strategies including increase in meal frequency, increase intake of energy dense foods, and use of oral nutritional supplements (14). However, there is lack of strong evidence in support of the effectiveness of dietary counselling in improving weight for cancer patients who are capable of oral intake [14]. Recently, there is a shift in nutritional oncology research from focusing on improving energy and protein intake to the provision of nutrition-related psychosocial support [15, 16]. Therefore, the multidimensional nature of nutritional problems occurring in the cancer patient points to the need for a more comprehensive approach to improve nutritional care for patients and their families.

The aim of this study was to evaluate the feasibility and acceptability of a patient- and family-centred intervention for improving nutrition intake among cancer patients using a different clinical focus and focusing on different aspects of the intervention in each site Recognising the impact of clinical context on such interventions we undertook this evaluation in two patient groups one who was receiving curative cancer treatments and the other palliative care.

## Methods

### Study overview

This PIcNIC study is building on a multi-faceted, family-centred nutrition intervention developed and tested in the context of critical illness [17, 18], refined for use in cancer patients at two different clinical settings (acute oncology ward & palliative care outpatient clinic) and cultural contexts (Australia & Hong Kong) (see Table 1). Due to the differences in settings, culture, and patient population, the study protocol was slightly adapted to each site (particularly around foods listed in the booklet; refer to ‘Intervention’ section below). The intervention incorporated principles of patient- and family-centred care (that is care that involves patients and families in the health care process, in our case the process of nutrition education, making appropriate nutritional choices to minimise nutritional impact symptoms and monitoring of nutrition intakes). The study was approved by the institutional review board of two universities (The HK Polytechnic University; and Griffith University in Australia (ref. 2016/200)) and hospitals in both countries (Hong Kong Kowloon Central/Kowloon East Research Ethics Committee, ref number KC/KE-16-0138/ER-2, and Gold Coast Health ref number HREC/16/QGC/75.

**Table 1:** Intervention protocol at each study site

Intervention details	Australia	Hong Kong
Delivered to:	Patient (and family member separately, if available)	Patient/family together
Delivered at:	Oncology ward	Patient’s home
Delivered by:	Dietitian	Dietitian
Intervention duration	5 to 7 days	4 weeks
Intervention components:		
Initial session (in person)	Brief nutrition history of the patient provided by patient/ family	
	Short, focused nutrition education/counselling session supplemented with a printed nutritional booklet	
	Introduction of a daily food record (AUS & HK version) to be completed by the patient/family	
	–	Negotiating nutritional goals
Follow up sessions	Reinforcement of nutrition education provided to patients and families prior to hospital discharge via face-to-face consultation	Reinforcement of nutrition counselling and adjustment of nutritional goals via telephone calls (at end of weeks 2 and 4 of intervention)
	Provision of a post-discharge nutrition plan	–
	Handover to outpatient dietitian upon hospital discharge for follow up (if required)	–

### Theoretical framework

The family systems theory served as the theoretical framework to guide the design of the study [19]. Its basic tenet is that a family system consists of interrelated individuals, each impacting the other and the family as a whole. Literature supports that a family member’s diagnosis of cancer can cause disruptions to stability of family functioning, with changing roles and relationships in both patients and family members [20]. According to family systems theory, the family is characterized as a goal-seeking and self-regulating system that functions in either adaptive or maladaptive ways [19].

### Study design and settings

The study was a mixed methods, interventional feasibility study, which incorporates multiple data sources from cancer patients, families and HCPs. The current paper presents data from the quantitative aspects of the study. This study was conducted in two hospitals, one in Australia [AUS] and one in Hong Kong [HK]. In Australia, data were collected in the inpatient oncology ward at Gold Coast University Hospital. In HK, data was collected from patients attending an outpatient palliative care unit of Haven of Hope Hospital.

### Sample

A convenience sample of 60 patients and their families and 20 HCPs were planned to be recruited, an equal number from each study site. ‘Family’ is defined for this study as any individual providing direct care to the patient on a regular basis, and also included domestic helpers in Hong Kong that are often providing care to patients at home. Selection criteria for patients, families and HCPs to participate in the study were:

Patient inclusion criteria:

- Age ≥ 18 years
- Diagnosed with solid tumour and receiving curative intent chemotherapy (AUS site) or having advanced cancer (stage IIIc–IV) and being off treatment (HK site);
- Life expectancy of ≥3 months, as judged by the treating physician;
- At risk of developing malnutrition as assessed by the Malnutrition Screening Tool with score ≥ 2 [21] or through dietitian assessment;
- Being capable of oral food and fluid intake;
- Able to communicate in English (AUS site) or Chinese (HK site);
- Had an family member actively involved in supporting the patient; and
- Able to provide informed consent.

Inclusion in the study based on the dietician’s experience and evaluation, even in the absence of a MST score of ≥2, was an approached tested in the AUS site, targeting patients with known high nutritional risk and nutrition impacting symptoms (ie. lung or gastrointestinal cancer patients). All patients in HK had a MST score of ≥2.

Patient exclusion criteria:

- Receiving enteral tube feeding, parenteral nutrition, dietary counselling or other types of nutritional interventions;
- Patients during the intervention who required a change in nutritional management that met exclusion criteria (i.e. initiation of parenteral nutrition) were excluded from continuing with the study and were recorded as drop-out cases.

Family selection criteria:

- Age > 18 years of age
- Able to communicate in English (AUS) or Chinese (HK);
- Expected to visit the patient regularly in hospital during their admission (AUS site) or at home (HK site).

HCP selection criteria:

Any nurse, physician, dietitian, and other allied health staff with experience in providing supportive care for cancer patients and who were caring for patients in the study (i.e. with exposure and familiarity to the intervention received by patients).

### Participant screening and recruitment

At the AUS site, research assistants conducted daily screening in the oncology ward to identify potential patient participants. Patients meeting eligibility criteria were approached and provided with study information, and informed consent was gained from those agreeing to participate. The families of enrolled patients were recruited if they visited patients regularly or provided care to patients at home. Following principles of Good Clinical Practice, in the morning the study was explained to the participants and opportunities to ask any questions were provided. The participants were then left to consider their option to participate. Researchers went back usually after lunch to see if they had further questions. If they agreed to participate in the study, their preferred time to receive the intervention was negotiated. Timing of intervention delivery was determined by the patient and it was often (but not always) the same day.

At the HK site, initial screening of potential patients and families was carried out by a home care nurse over the phone before the day of medical consultations. When attending the clinic, patients and families meeting the eligibility criteria and showing interest in the study were referred to a research assistant and were provided information about the study. Informed consent was obtained from those participants agreeing to participate. The intervention was delivered within a two-week period following consent.

### Intervention

The intervention was a patient- and family-centred nutritional education program designed for improving energy/protein intakes of cancer patients. The intervention was premised on providing nutrition education and counselling and monitoring nutrition intakes. There were two key components to the intervention; education and monitoring dietary intake. The education component incorporated face-to-face education which was supplemented by printed material. The development of the educational booklet (see Additional file 1) was informed by the literature, our previous research, and the clinical teams’ expertise in nutrition, similar to such booklets from various organisations. The booklet contained information on the importance of nutrition therapy during cancer treatment, approaches to support nutritional intake, management of nutrition-impacting symptoms, dealing with eating- and weight-related distress, and practical tips for patients and caregivers. Monitoring of dietary intake was done using a 3-day paper food record that was completed by the patient and/or family member (or assessment by the nutritionist in the AUS site). Food records involved documenting amounts of food, fluids and any nutrition supplements consumed by the patient at each meal. Both the education provided and the documentation of nutrition intake was adapted to the site requirements. More specifically, in addition to the common information covered in the AUS booklet, the HK booklet added the elements of psychosocial aspects of nutritional care [21] and practical nutrition-related skills for patients and family to explore the feasibility of such an additional component (although the AUS site also incorporated these components to a lesser degree). Essentially the two sites were testing different aspects of an intervention (ie. short delivery vs longer delivery; patients receiving anticancer treatment vs advanced cancer patients; intervention delivered in hospital vs at home; acute setting vs palliative care setting; fewer intervention components vs adding psychosocial elements and goal setting, etc), in order to explore which intervention is more suitable for further testing in the future, if it was found acceptable and feasible. The content of the food record was adjusted to reflect the dietary habits of Chinese population in the HK site.

Tailoring of the intervention at each site was required to meet patient specific requirements. Patients with advanced cancer who were off treatment (the HK site) also had guided nutritional-related goal setting and dietary adjustments incorporated as part of the intervention; both align with two important features of Family Systems Theory: goal-seeking and self-regulation [20]. During the intervention period, patients and families were supported to develop action plans for implementing goal-oriented dietary behaviours. Goals were usually specific and quantifiable, explained in a language understandable by the patient/family. Patients and families were given two weeks to perform goal-oriented nutritional support behaviours, before being contacted again to evaluate how well goals were met or re-adjust the goals. This required them to master the relevant knowledge and skills and learn to negotiate and communicate on decisions made in terms of when, how often and what to eat.

Details on what the intervention involved at each site are shown in the Table 1. The intervention was delivered by trained dieticians, who had experience in working with cancer patients and had a minimum of a Bachelor’s degree in dietetics. The dieticians received additional training on the trial processes and the delivery of the intervention.

### Outcome measures

Primary outcomes related to the feasibility and acceptability of and adherence to the intervention.

#### Feasibility

Feasibility data included eligibility, recruitment and retention rates; and fidelity of intervention delivery, which were collected through screening, recruitment, retention and intervention delivery logs. Feasibility criteria were as follows:

- Eligibility: ≥50% of patients and families within the study setting meet eligibility criteria;
- Recruitment: ≥80% of eligible patients and families consent to participate in the study;
- Retention: ≥80% of recruited patients complete the study; and
- Fidelity of intervention delivery: ≥80% of participants receive the full intervention.

#### Acceptability

Acceptability was measured through quantitative surveys of patients’, families’ and HCPs’ perceptions of and satisfaction with the intervention. Surveys were tailored to each participant group (i.e. patient, family, HCP) and were completed 1–2 weeks following intervention completion, or earlier if the patient was discharged from hospital.

#### Adherence to the intervention

This was assessed through evaluation of food records completed by patients/families during the intervention and at how many patients had the full intervention delivered.

#### Nutritional indicators

Secondary outcome data included patients’ estimated (by the dietitian) energy and protein intakes. The calculations used to estimate energy and protein requirements were based on Liu’s equation of basal metabolic rate x activity factor specific for Chinese patients [22].

### Data collection

Demographic data pertaining to the patient (age, gender, cancer diagnosis, treatments, comorbidities, height, weight, body mass index, MST score, PG-SGA score [23], previous dietetic input), their family (age, gender, relationship to patient, employment status, education level) and HCPs (age, gender, position, highest qualification, years’ experience) were collected through chart audits or in surveys.

### Data analysis

Data was entered and analyzed using IBM SPSS 21.0 software. Descriptive statistics were used to summarize sample characteristics, feasibility, acceptability and nutritional related data, including mean, standard deviation, median, intra-quartile range, and percentages. Food chart completion was analysed quantitatively by determining the number/amount of meals recorded by patients/families. Patients’ energy and protein intake was estimated by the dietitian through nutritional history taking and comparing the baseline with the post-intervention levels using Friedman’s test.

## Results

### Hong Kong data

A total of 30 patients and 30 family members consented to participate in the study. The demographic and clinical characteristics of patients are detailed in Table 2. Twenty-one patients and 19 family caregivers completed the surveys. The caregivers were predominantly spouses/partners (37%) and children (47%); female (74%); and > 40 years old (75%) while most of them were unemployed or retired and received less than high school education.

**Table 2:** Patient characteristics (*N* = 53)

Characteristics	HK site (*N* = 30)	AUS site (*N* = 23)
	Mean ± SD (range)	Mean ± SD (range)
Age (years)	73 ± 13 (53–97)	54 ± 18 (18–79)
BMI (kg/m^2^)	20.6 ± 3.7 (14.6–29.3)	29.2 ± 7.2 (19.4–46.8)
	N (%)	N (%)
Gender		
Female	17 (56.7)	11 (47.8)
Male	13 (43.3)	12 (52.2)
Cancer type^a^		
Lung	9 (30.0)	7 (31.8)
Colon	8 (26.7)	3 (13.6)
Prostate	3 (10.0)	1 (4.5)
Liver	3 (10.0)	0
Cervical	2 (6.7)	2 (9.1)
Breast	0	5 (22.7)
Others	5 (16.5)	2 (9.0)
Co-morbidities		
No	9 (30.0)	5 (21.7)
Yes	21 (70.0)	18 (78.3)
Liver disease	7 (23.3)	1 (4.3)
Diabetes	5 (16.7)	0
Pressure injury	2 (6.7)	0
Chemotherapy or radiation	2 (6.7)	12 (52.2)
Head injury	1 (3.3)	
Chronic renal failure	1 (3.3)	2 (8.7)
Chronic obstructive pulmonary disease	1 (3.3)	4 (17.4)
Infection	1 (3.3)	7 (30.4)
Surgery	0	1 (4.3)
MST score ^b^		
0	0	7
1	0	3
2	21 (70)	
3	7 (23.3)	
4	2 (6.7)	1
5		1
PG-SGA-SF		
> 3 (malnutrition threshold)	25 (83.3)	
PG-SGA or SGA score^c^		
A		11 (52.3)
B		9 (42.9)
C		1 (4.8)

^a^*n* = 22 (AUS site); ^b^*n* = 12 (AUS site); ^c^ *N*= 21 (AUS site)

Ten HCPs were also recruited, including doctors (20%), dietitians (20%), and nurses (60%). Most HCPs were female, < 40 years old, with >five years of clinical experience (55%).

### Feasibility

**Fig. 1:**
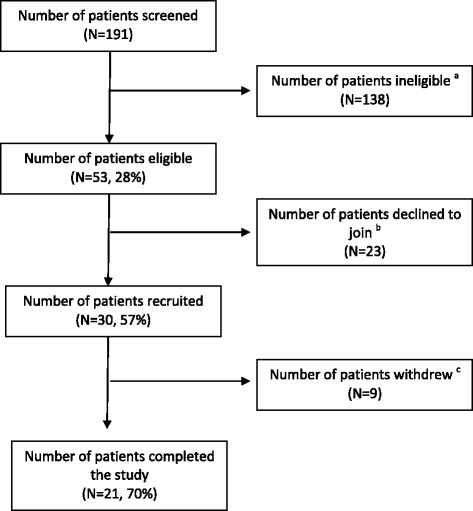
Flow chart of patient screening, recruitment and retention (HK site). ^a^Reasons for not meeting eligibility criteria: 1) Absence of a family caregiver (*n* = 73); 2) patient not living at home(*n* = 52); 3) family unable or unwilling to provide regular nutrition support (*n* = 5); 4) incapable of oral intake (*n* = 3); 5) family caregiver unable to speak Chinese (*n* = 3); and 6) patient life expectancy less than 3 months (*n* = 2). ^b^Reasons for declining to join the study: 1)patient/family felt intervention unnecessary (*n* = 6); 2) patient/family felt that the intervention might not help them (*n* = 3); 3) family having no time to participate (*n* = 2); 4) patient/family dislike home visit (*n* = 2); 5) patient too stressful about disease (*n* = 2); 6) patient/patient having no interest (*n* = 2); 7) Patient too old(*n* = 1); and 7) unknown reasons (*n* = 5). ^c^Reasons for withdrawal: 1) re-hospitalization (*n* = 4); 2) death (*n* = 3); 3) family matter (*n* = 1); and 4) not reachable via the phone (*n* = 1)

We recruited palliative care cancer patients and their family caregivers as planned. Feasibility criteria were not met according to the predetermined criteria (Table 3). After screening 191 patients, 30 patients and 30 caregivers were recruited to the study from a total of 53 patients that were eligible to participate based on the study criteria, yielding the recruitment rate of 57%. Of 30 patients, nine did not complete the study due to patient re-hospitalization, death or other family matter. The retention rate was 70%. The rest of 21 patients completed all components of the intervention, and the fidelity rate was 70% (Fig. 1).

**Table 3:** Feasibility data

Indicators	Predetermined criteria	AUS	HK
Eligibility rate	≥50%	19%	28%
Recruitment rate	≥80%	23%	57%
Retention rate	≥80%	65%	70%
Fidelity rate	≥80%	35%	70%

### Acceptability

As can be seen in Table 4, survey data from patients and family caregivers indicated high acceptability of the intervention. Most patients and caregivers found the intervention informative in terms of improving their nutrition knowledge. Between either nutritional counselling or the booklet alone, > 80% of patients and caregivers also agreed the combination is best. Although most family members and patients used the booklet either ‘never’ or ‘rarely’, the information in the booklet was easy to understand for most participants. The food charts were fairly easy to complete for over half the patients and families. Paper versions, as opposed to electronic ones, were the preferred method for completing food charts in the vast majority (> 80%). When asked if the intervention had positive impact on them, most patients and families responded “yes” and would participate in the future in a similar study.

**Table 4:** Selected acceptability data from patients and family caregivers at each site^a^

	Response	AUS patients (*N* = 14)	AUS family (*N* = 3)	HK patients (*N* = 9)	HK family (*N* = 19)
		N (%)	N (%)	N (%)	N (%)
The nutrition counselling & booklet provided new information	Yes	12 (86)	0 (0)	8 (89)	16 (84)
I was comfortable participating in the study	Comfortable/very comfortable	5 (36)	0 (0)	7 (78)	17 (90)
The booklet was easy to understand	Fairly easy/very easy	11 (85)	0 (0)	6 (67)	17 (90)
The food intake chart was easy to use	Fairly easy/very easy	11 (79)	2 (67)	5 (56)	10 (53)
I was satisfied with the nutrition care received	Satisfied/very satisfied	11 (79)	0 (0)	8 (89)	17 (90)
Impact of intervention on you/family:	Positive impact	8 (62)	0 (0)	6 (67)	12 (63)
	No impact	5 (39)	0 (0)	3 (33)	7 (37)
I would participate in a similar study in the future	Likely/very likely	10 (71)	3 (100)	7 (78)	10 (53)

^a^All acceptability items show similar trends and are available by the authors upon request

Many patients and family caregivers provided a significant number of comments on the intervention, written as additional comments in the quantitative questionnaire. These were about focusing on dietary preferences, preparation of food particularly in those experiencing dysphagia, and improving food intake records. Patients and caregivers reported that they felt cared for through this intervention, increased their nutritional intake and knowledge on food, and enhanced communication with the family over food. However, some also highlighted that nutritional needs differ from patient to patient and that it may be appropriate to offer suggestions for special diets depending on the symptoms they are experiencing. Key nutrition-impacting symptoms reported by patients included fatigue (82%), dry mouth (68%), lack of appetite (64%) and pain (57%). A few found it difficult to adhere to the new knowledge and did not change their eating, due to barriers such as high costs, issues with accessing some food items, or cooking in a different way than usual.

In terms of HCPs’ views, 90% agreed/strongly agreed that they support the notion of families partnering with HCPs to achieve optimal patient nutrition. In terms of who should determine how much information patients need about their nutritional status, 90% felt it should be the family, 70% the nurses, 40% the doctors, and 100% the dieticians. All HCPs agreed/strongly agreed that patients and families should be encouraged to participate in nutrition care. All HCPs also agreed that patients/families should be able to discuss their concerns about nutrition with HCPs and agreed that the intervention improved the patients’ nutritional status somewhat (70%) or a lot (10%).

Some insights into the intervention from HCPs were reported. Four cited the presence of a competent caregiver with adequate nutritional knowledge as crucial in the success of the intervention. However, half of the HCPs also cautioned that some advanced cancer patients with multiple symptoms and psychological distress may not prioritise enough nutrition in their care. Five HCPs mentioned that organizational factors, such as time and manpower are considerable barriers to the implementation of such an intervention in clinical settings.

### Food chart completions

Data was collected on the adherence to return and completion of food intake charts at two time points; the end of weeks 2 and 4. At the first assessment point, 25 of 26 (96.2%) patients/family caregivers completed food records and returned them to the research team. For the second assessment points, 22 of 23 returned food records (96%) and 20 completed them (91%) as the other two food records were returned but not completed. Out of the 30 participants, 21 completed the full intervention (70%), although 19 patients provided final data on all outcomes 1–2 weeks after the end of the intervention (63%).

### Nutritional intake/nutritional status

Clinical outcome data showed an improvement in all variables of energy and protein requirements and intake, as shown in Table 5.

**Table 5:** Changes in clinical outcomes (HK data only)

Variables	Baseline	1st day of week 3	1st day of week 5
	Median (IQR)	Median (IQR)	Median (IQR)
	*N* = 28	*N* = 24	*N* = 21
%EER^1^	80 (61–93)	97 (71–103)	96 (87–106)
% EPR^1^	82 (60–98)	92 (78–129)	93 (82–117)
Energy intake (kcal/kg/day)	22 (17–26)	24 (17–32)	26 (24–32)*
Protein intake (g/kg/day)	0.9(0.7–1.1)	1.0(0.8–1.4)	1.0(0.9–1.3)**

*IQR* Interquartile range, *EER* Estimated energy requirements, *EPR* Estimated protein requirements; ^1^(numbers indicate median % EER/EPR met across patients)

*p = 0.01 (Friedman test); **p = 0.013 (Friedman test)

### Australian data

Twenty-three patients and three family members consented to participate in the study (see patient characteristics in Table 2). Twenty HCPs were also recruited, including doctors (50%), dietitians (5%), and nurses (45%).

### Feasibility

**Fig. 2:**
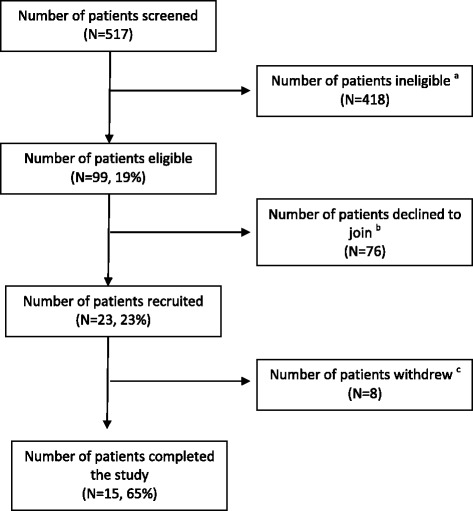
Flow chart of patient screening, recruitment and retention (AUS site). ^a^Reasons: not meeting eligibility criteria (*N* = 325): 2) poor physical and mental status (*n* = 93). ^b^Reasons for declining to join the study: discharged prior to being approached for consent (*n* = 8); others including patient expecting to be discharged soon, patient already seen by a dietitian, being overwhelmed with current admission (*n* = 68). ^c^Reasons for withdrawal: 2 patients died, 1 patient transferred to another hospital, 1 patient refused to complete food diary and survey, 1 patient couldn’t remember intervention and 1 patient deteriorated medically

Recruiting family caregivers was more challenging than anticipated at the AUS site as they often did not accompany the patients at the time of recruitment or they were not contactable, often being at work. Feasibility criteria were not met (Table 3). In this site (Fig. 1), despite screening over 500 patients, only a small proportion of patients in the oncology unit were eligible to participate in the study (19%) with patients too bus or concerned with their treatment delivery primarily and nutrition was less of a priority at the time, and of these, only 23 patients agreed to participate and were recruited (23%). Only 15 patients completed the study (65%), alongside three family members (two spouses and one parent, all female) (Fig. 2).

### Acceptability

Survey data from patients also indicated high acceptability of the intervention in this site. Similar responses reported in the HK site were reported here too. Between either nutritional counselling or the booklet alone, > 80% of patients also agreed the combination is best. The food charts were fairly easy to complete for two-thirds of the patients. Paper versions, as opposed to electronic ones, were the preferred method for completing food charts in the vast majority in this site too. When asked if the intervention had positive impact on them, most patients also responded “yes” and would participate in the future in a similar study.

In terms of HCPs’ views, all HCPs agreed/strongly agreed that they support the notion of families partnering with HCPs to achieve optimal patient nutrition. In terms of who should determine how much information patients need about their nutritional status, 35% felt it should be the family, 15% the nurses, 30% the doctors, and 65% the dieticians. 85% of HCPs agreed/strongly agreed that patients and families should be encouraged to participate in nutrition care. Almost all (95%) also agreed that patients/families should be able to discuss their concerns about nutrition with HCPs.

## Discussion

This study assessed the feasibility and acceptability of a patient- and family-centred psychosocial and nutritional-based intervention for improving nutrition among oncology patients. According to our pre-determined criteria, the study was not feasible as eligibility, recruitment and retention targets were not met, perhaps reflecting the complexity of the treatment setting and the ill-health status of the population. The intervention was more appropriate for advanced cancer patients at the home care setting, meeting adherence targets, fidelity and with acceptable recruitment and retention rates (albeit lower than the initial targets), considering the patients’ condition and complexity of symptoms experienced during the intervention. Also, the intervention was relatively easy to deliver and was found to be acceptable by patients, families and HCPs. It may also have the potential to improve management of nutritional intake and symptoms.

At the AUS site, there were issues around recruiting family members in the acute oncology setting, due to multiple logistical issues and family members not being present within the working hours of the dietitians, who delivered the intervention. The Australian site also targeted patients (ie. with lung or gastrointestinal cancer) who had not necessarily nutritional problems (but were at high risk of developing such problems, being cancer diagnostic groups with often high prevalence of malnutrition and nutrition impacting symptoms). Thismay have contributed to low recruitment as patients may not have found participation to this trial relevant or useful for them. Also, the Australian site had, alongside better nutritional status, a briefer intervention period and patients generally younger than the HK site. Recruitment was feasible in the palliative care context (HK), however only as a home care study. The focus on home delivery of the intervention was decided early in the study after recognising the difficulties in delivering the intervention to this population in a palliative care ward. Challenges in recruiting general cancer as well as palliative care patients into trials are well-reported in the literature and different ways of recruiting patients more successfully have been explored in the past [24, 25]. Although recruitment rates did not reach the pre-determined target of 80%, the 70% recruitment rate achieved was deemed appropriate considering the complex condition of advanced cancer patients. Hence, we concluded that the study is feasible primarily in the context of advanced cancer, where nutritional issues are also more prominent, and in the home setting.

Interestingly, patients, families and HCPs who were involved in the study found it acceptable in that they were satisfied with it, perceived benefits from participating, and would welcome similar interventions in the future. Previous studies suggest that patients and families want to be actively involved in their health care in both hospital and home setting [26, 27]. Families of oncology patients may feel helpless and unable to contribute in many ways due to complexity of treatments, however food/nutrition is an area families are likely to feel comfortable with [11]. Patients and families have the ability to meaningfully contribute to their health care and outcomes [28].

In addition to dietary counselling and self-management education, this intervention also focused on managing malnutrition-related distress, which is common in advanced cancer patients [29]. This was done by providing nutritional advice in a more flexible way in terms of what to eat, improving communication between the patient and family member, addressing individual needs and concerns, and setting up achievable goals. Managing malnutrition-related stress was not only targeted to patients, but also to family members, as we know from the literature that families of advanced cancer patients often experience (mal)nutrition-related distress and often need nutritional knowledge and support to care more effectively for their loved members [11, 30]. It is also clear that family caregivers have unmet needs in dealing with the patient’s eating problems and would benefit from education and support [31]. The potential of the family caregiver to be an important agent in the management if weight loss and anorexia in advanced cancer has been empirically supported in the past in small scale feasibility studies [32, 33], an area that merits further investigation. Also, outcome measures should focus on the family’s eating-related distress and self-efficacy in managing the patient’s nutritional problems.

Energy and protein intakes relative to estimated energy and protein requirements had significant improvement over the duration of the study, with patients’ intakes increasing over time (HK site). However, the small sample size and crude estimation of nutritional intakes (estimated from patient/family-reported food diaries) limits the accuracy and applicability of this data, and only serves as an indication of the potential effectiveness of the intervention. These promising findings in energy and protein intake are supported by other studies, where significant correlations were observed between dietary energy/protein intake and weight change in advanced cancer [34], although what is not so clear from the literature is whether this alone is sufficient to maintain or increase body weight and other objective measurements of nutritional status [14, 34]. A larger trial with an adequate sample size, a control group, and appropriate measurement techniques is required to make any judgement about the effectiveness of the intervention on patients’ nutritional intakes. This study is consistent with other studies of nutrition in cancer, with improvements in nutritional intake observed in response to nutritional counselling. Similar improvements have been seen in other nutritional trials in the past, including an interdisciplinary nutrition-rehabilitation programme, particularly around nutrition impact symptoms [35], intensive dietary counselling over standard dietary counselling [36] or individualised nutritional therapy [37], suggesting that individualised and more intensive approaches in advanced cancer patients can lead to nutritional improvements. In addition to nutritional intakes, psychosocial-based nutritional outcomes would also be appropriate outcome measures for a future trial. Based on the comments made by both patients and family members, outcomes should also focus on nutrition-related communication and/or distress, and self-efficacy in managing the patients’ nutritional problems.

Key nutrition impacting symptoms were present in patients and, while some information was directed to them in the booklet, this is an area that participants wanted more specific information and guidance on what to do when the patients experience certain symptoms. Also, improvements in nutrition impact symptoms could be another set of outcomes in a future trial.

### Study limitations

This study has several limitations. Firstly, the intervention targeted advanced cancer patients with relatively high performance status and capable of oral intake. Those at end of life or with complex nutritional needs (who may have benefited from nutritional intervention) were not included as they necessitate different nutritional management. Furthermore, as the intervention focused on the family pair, it is possible that individual patients who did not have a carer/family member available may have also benefited from this intervention, but did not meet eligibility criteria. The response rate for the acceptability questions was low, particularly in HK patients, as many of them were unwell and their condition was becoming worse, preventing them from completing the questionnaires; this also reflects the realities of research in palliative care setting. A future intervention may have to be adjusted to meet the needs of such patients. Secondly, patients and/or family members who were more interested in nutrition may have been more willing to participate in the study and more likely to find it acceptable. Participants also made a number of suggestions to improve the intervention itself; for example, they wanted more tailored counselling on managing swallowing difficulties; which will be incorporated into the future intervention plan. Future trials should consider factors that may impact on the success of a nutritional intervention outcomes such as age, functional status, current anticancer treatment, presence of symptoms (particularly loss of appetite or mucositis), number of drugs used by the patient or type of cancer [34, 38, 39].

## Conclusions

This study provides initial feasibility data of a new intervention, suggesting that providing nutritional family-based education to patients with advanced cancer and their families supplemented by nutritional psychosocial support may be a useful way to enhance the patient-family member dyad’s nutrition-related communication, decrease distress from eating difficulties and possibly can improve nutritional clinical outcomes. Using the lessons learnt from this feasibility work, it is clear that the intervention was helpful to patients and families, hence the next step would be to develop a pilot randomised trial using a refined intervention in advanced cancer patients and assess the process of the trial, measures that could be appropriate and sensitive for use in a larger trial and have early data to allow for sample size calculations for the larger trial. Refinement of the new trial includes the content of the booklet as commented by patients and families, the assessment of nutritional intake, the inclusion of patients who do not have a caregiver, the enhancement of the nutritional psychosocial element of the intervention, and incorporation of caregiver assessment on family communication and caregiver distress. For future research, as food and eating are culturally-specific concepts, nutritional interventions need to be tested in different countries/cultures to assess how applicable they are in a given context.

## Additional file

Additional file 1: Nutrition booklet. (PDF 531 kb)

## Abbreviations

AUS: Australia
HCP: Health care professional
HK: Hong Kong
MST: Malnutrition Screening Tool
PG-SGA: Patient generated-Subjective global assessment
PIcNIC: Study acronym; Partnering with families to promote nutrition in cancer care
