# Supplementary material for: Partnering with families to promote nutrition in cancer care: feasibility and acceptability of the PIcNIC intervention
Source: BMC Palliat Care. 2018 Mar 20;17:50. doi: 10.1186/s12904-018-0306-4 (PMC5859412; doi:10.1186/s12904-018-0306-4)

Nutrition in cancer care

Educational booklet for patients and families

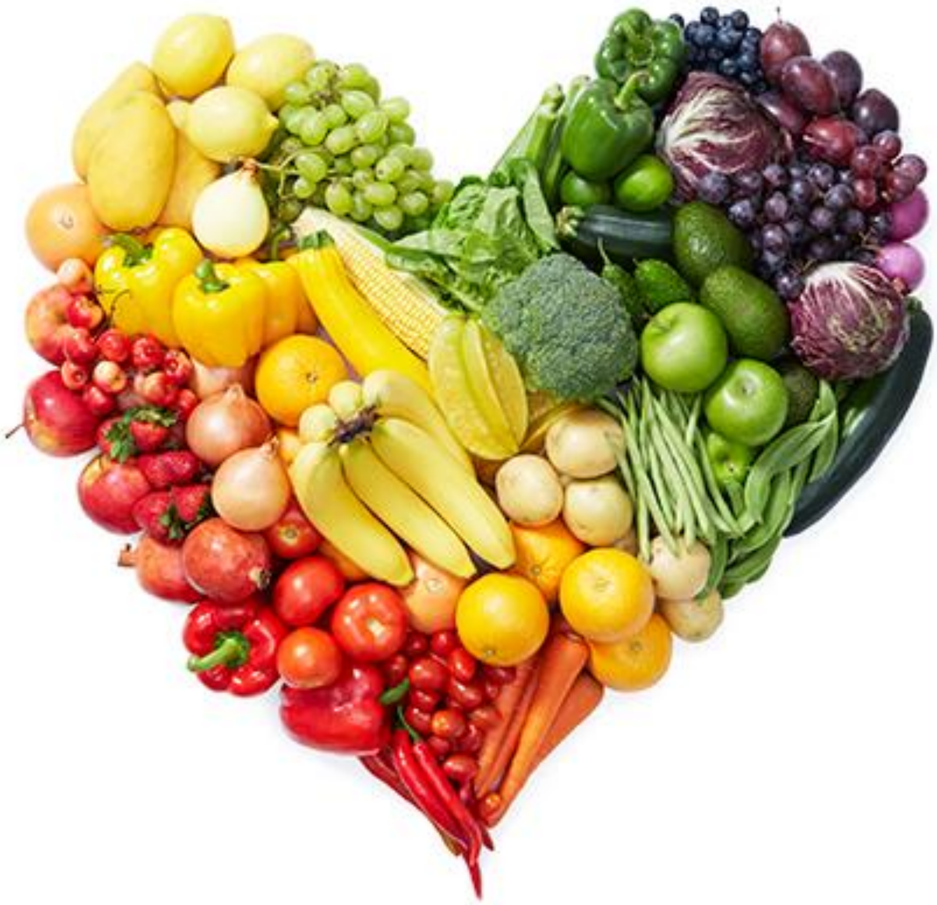

This booklet contains information about:

- The importance of nutrition as part of your treatment
- How we can support nutrition intake
- Common problems affecting nutrition and how to manage these
- Things you can do to help your family member with nutrition

The information contained in this booklet has been prepared by:

Professor Andrea Marshall

Professor of Acute and Complex Care Nursing, Griffith University & Gold Coast Hospital and Health Service

Jessica Abbott

Senior Dietitian – Oncology

Gold Coast Hospital and Health Service

Dr Shelley Roberts PhD

Research Fellow – Griffith University, Gold Coast

Clinical Dietitian, Gold Coast Hospital and Health Service

Professor Liz Isenring

Head of Faculty of Health Services and Medicine

Bond University, Gold Coast

Professor Alex Molasiotis

Angel S.P. Chan Lau Professor in Health and Longevity, Hong Kong

Chair Professor of Nursing and Head Director of WHO Collaborating Centre for Community Health Services, Hong Kong.

Nutrition as part of your treatment

Nutrition is essential for health and wellbeing, especially during illness. Good nutrition can help tissues and wounds to heal better; prevent problems caused by not eating or drinking enough; and even shorten hospital stay. As such, it is considered a therapy or treatment just like medications.

Nutrition therapy means giving people the correct amount and type of nutrition for their needs. For patients with cancer, this helps to:

- Prevent unwanted weight loss during treatment
- Maintain muscle, energy, strength and mobility
- Support the immune system
- Promote healing of damaged tissues during and after cancer treatment
- Help cope with side-effects of some treatments
- Reduce risks of complications like infections, pressure ulcers or falls in hospital

How can we support nutrition intake?

Nutrition may be delivered in a number of ways, including:

- Oral nutrition – foods and fluids are consumed by the mouth
- Tube feeding – special liquid nutrition formula given through a tube that goes into the stomach or bowel
- Intravenous (IV) nutrition – specialised nutrition given directly into the blood stream
- A combination of any of the above

Oral nutrition is the preferred and most common method for providing nutrition. It includes:

- Regular foods
- Texture modified foods (e.g. softer, smooth foods)
- Food fortification (e.g. adding something extra into foods to boost nutrition content)
- Specialised diet plans (e.g. high protein, high energy)
- Nutrition supplements (e.g. high protein drinks).

Tube feeding is used when a patient is not able to eat and drink enough food and fluids through the mouth. Patients may receive all or just some of their nutrition this way. The tube may be inserted into the stomach through the nose or may pass directly into the stomach through an incision made in the skin (PEG tube). The dietitian working with the medical team will prescribe the correct specialised nutrition formulas for the patient.

Intravenous (IV) nutrition is given by a thin tube going into a vein. IV feeding is the least common type of nutrition therapy, but may be used if the stomach or bowel is not working properly, or the patient is not tolerating tube feeding.

If you are concerned at any time that your family member is not receiving enough nutrition, please ask to speak with the nurse or dietitian.

Common problems affecting nutrition

During hospital stays, it can be difficult for patients to meet their nutritional needs. Patients undergoing treatment for cancer may have higher nutritional needs and experience problems that affect their food intake.

During cancer treatments such as surgery, chemotherapy, drug therapy and radiation, the body uses more energy (calories) and protein. So, the body needs more energy and protein than usual. Some treatments can also have side-effects which can make eating and drinking difficult, so it is harder for patients to meet these increased needs.

Common side-effects that can impact eating and drinking include:

- Loss of appetite
- Nausea and/or vomiting
- Mouth or throat ulcers
- Disrupted bowel habits (i.e. diarrhoea or constipation)
- Taste and smell changes

The table below suggests some strategies that may be useful in helping you consume enough nutrition if you are experiencing these problems.

Strategies to improve your nutrition intake

Symptom	Strategies to help
Loss of appetite	• Eat smaller amounts more often<br>• Drink fluids after or between meal times<br>• Eat items that provide the most energy and protein first (e.g. meat, eggs, chicken, fish and dairy foods)<br>• Distract yourself: you may eat more when you're not thinking about it – try focusing on the TV or chat to family or friends<br>• Keep moving: exercise stimulates appetite and digestion<br>• Think of food as your fuel and part of your treatment
Nausea and vomiting	• Eat regularly to prevent hunger, which can worsen symptoms<br>• Try cold clear fluids like juice, ginger ale and lemonade<br>• Choose 'cold' foods from hospital menu (e.g. sandwiches, desserts)<br>• Make the most of when you are feeling well by consuming energy-rich foods and fluids
Mouth or throat sores	• Select softer, smoother food items from the hospital menu. Ask for a softer texture or smooth diet if you need to<br>• Cold foods and fluids like ice cream, yoghurt, custard, milks and other cold drinks can be soothing and also provide nutrition
Diarrhoea	• Drink plenty of fluids to replace extra losses through diarrhoea<br>• Limit caffeine in tea, coffee and some soft drinks (e.g. Cola)<br>• Sometimes soluble fibre (e.g. Metamucil, apple sauce) can help
Constipation	• Drink plenty of fluids to keep stools soft<br>• Increase fibre intake with wholegrain cereals, fruits and vegetables<br>• Keep moving – exercise helps with digestion
Taste and smell changes	• Experiment with different foods on the menu as some may taste better than others. Try unusual combinations. Sometimes a sweet sauce goes well with savoury foods.<br>• Ask for plastic cutlery if metal cutlery causes a 'metallic' taste<br>• Try drinking through a straw to by-pass some of the tastebuds

Things you can do to help your family member with nutrition

- Encourage your family member with their eating and drinking. Remember, every mouthful counts.
- Make mealtimes an enjoyable and social occasion – you may wish to eat together at some mealtimes in the hospital.
- Ask your family member about their nutritional intake. Questions you may wish to ask include:
  - Are you eating less than normal?
  - Are you eating less than half of your meals?
  - Do you have any symptoms making it difficult for you to eat?
- Ask the health professionals (e.g. doctor, nurse, dietitian) if your family member is getting enough nutrition. Questions you may wish to ask include:
  - How much should they be eating and drinking each day?
  - Are any side-effects stopping them from eating enough?
  - How much food are they eating from each meal tray?
- Bring in your family member's favourite or preferred foods or drinks. Ask the nurse or dietitian what foods are okay to bring in.

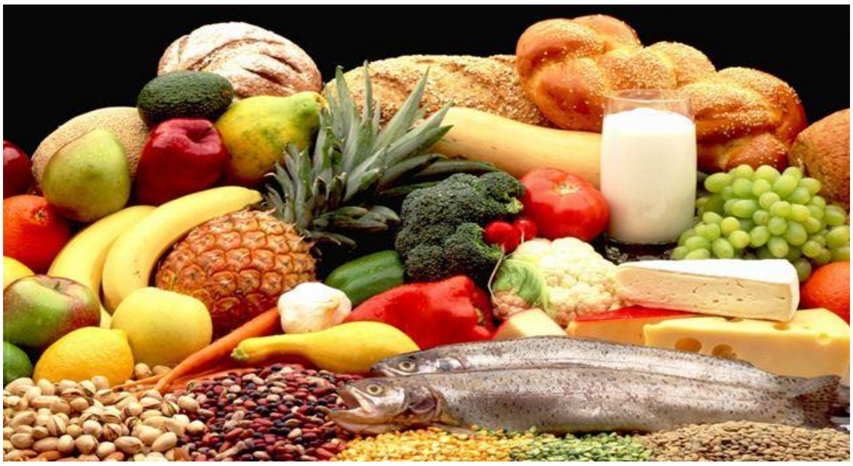

If you are concerned about your family member's nutrition whilst in hospital, you may ask to see a dietitian for advice.

Other useful resources: Cancer Council *'Nutrition and Cancer'*

Available at: https://cancerqld.org.au/wp-content/uploads/ccq-resources/Nutrition%20and%20Cancer.pdf or contact the Cancer Council Helpline on 131120

If you have any questions about the PICNIC study, you can ask to talk with the researcher leading this study at Gold Coast University Hospital.

Researcher's name:	Professor Andrea Marshall
Phone number:	(07) 5687 3235
Email:	a.marshall@griffith.edu.au

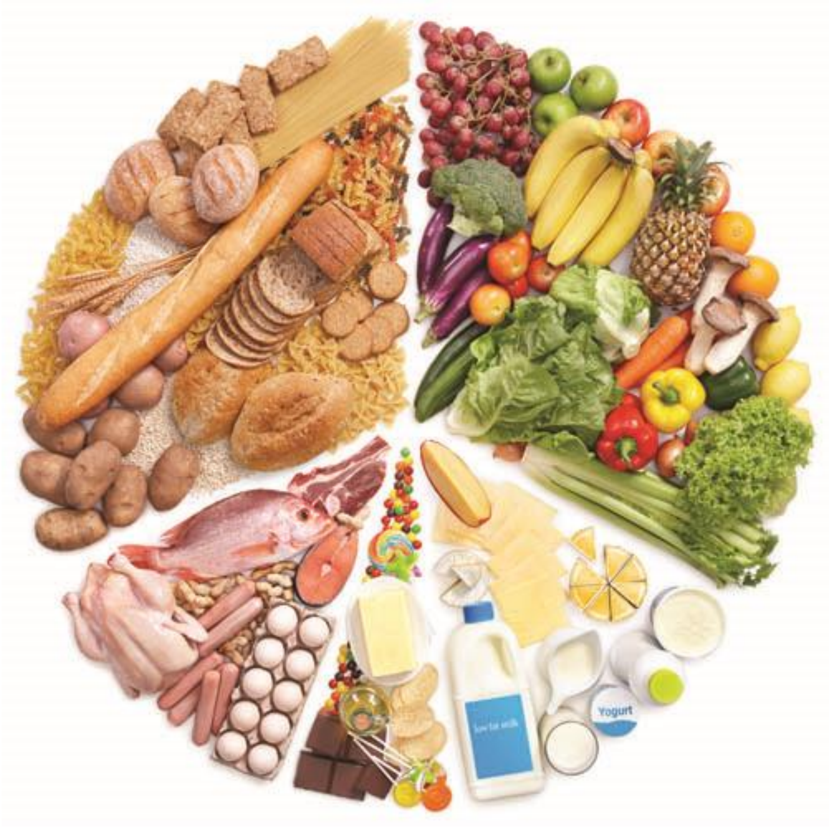

Supplement: Supplementary file 1 — Nutrition booklet. (PDF 531 kb) [file 12904_2018_306_MOESM1_ESM.pdf]
